# Addition of gadolinium contrast to three-dimensional SSFP MR sequences improves the visibility of coronary artery anatomy in young children

**DOI:** 10.3389/fped.2023.1159347

**Published:** 2023-05-05

**Authors:** Quanli Shen, Chengxiang Lin, Qiong Yao, Junbo Wang, Jian Zhou, Lan He, Gang Chen, Xihong Hu

**Affiliations:** ^1^ Department of Radiology, Children’s Hospital of Fudan University, Shanghai, China; ^2^ Heart Centre, Children’s Hospital of Fudan University, Shanghai, China

**Keywords:** magnetic resonance coronary angiography, whole heart, steady-state free precession, contrast media, children

## Abstract

**Objective:**

This study aims to compare the value of a gadolinium contrast-enhanced 1.5-T three-dimensional (3D) steady-state free precession (SSFP) sequence with that of a noncontrast 3D SSFP sequence for magnetic resonance coronary angiography in a pediatric population.

**Materials and methods:**

Seventy-nine patients from 1 month to 18 years old participated in this study. A 3D SSFP coronary MRA at 1.5-T was applied before and after gadolinium-diethylenetriaminepentaaceticacid (DTPA) injection. The detection rates of coronary arteries and side branches were assessed by McNemar's *χ*^2^ test. The image quality, vessel length, signal-to-noise ratio (SNR), and contrast-to-noise ratio (CNR) of the coronary arteries were analyzed by the Wilcoxon signed-rank test. The intra- and interobserver agreements were evaluated with a weighted kappa test or an intraclass correlation efficient test.

**Results:**

A contrast-enhanced scan detected more coronary arteries than a noncontrast-enhanced scan in patients under 2 years old (*P* < 0.05). The SSFP sequence with contrast media detected more coronary artery side branches in patients younger than 5 years (*P* < 0.05). The image quality of all the coronary arteries was better after the injection of gadolinium-DTPA in children younger than 2 years (*P* < 0.05) but not significantly improved in children older than 2 years (*P* > 0.05). The contrast-enhanced 3D SSFP protocol detected longer lengths for the left anterior descending coronary artery in children younger than 2 years and the left circumflex coronary artery (LCX) in children younger than 5 years (*P* < 0.05). SNR and CNR of all the coronary arteries in children younger than 5 years and the LCX and right coronary artery in children older than 5 years enhanced after the injection of gadolinium-DTPA (*P* < 0.05). The intra- and interobserver agreements were high (0.803–0.998) for image quality, length, SNR, and CNR of the coronary arteries in both pre- and postcontrast groups.

**Conclusion:**

The use of gadolinium contrast in combination with the 3D SSFP sequence is necessary for coronary imaging in children under 2 years of age and may be helpful in children between 2 and 5 years. Coronary artery visualization is not significantly improved in children older than 5 years.

## 1. Introduction

The global incidence rate of coronary artery abnormalities is about 1%–5.6%, and the incidence in patients with congenital heart disease (3%–36%) is higher (1). When there is a clinical imperative to image coronary artery anatomy, the choice of cross-sectional modalities, such as echocardiography (ECHO), computed tomography (CT), magnetic resonance imaging (MRI), and the gold standard catheter angiography, depends on the detail of information required weighed against the use of ionizing radiation, need for sedation, and degree of invasion (2–8). Magnetic resonance coronary angiography (MRCA) is widely used for relatively gross imaging of coronary arteries, with extensive use now being made of cardiac and respiratory-gated (free breathing), T2-prepared, three-dimensional (3D) steady-state free precession (SSFP) sequences (6, 9–15). These sequences have a vital application in children, particularly those in whom ongoing follow-up of the coronaries is planned due to their lack of ionizing radiation, but have been limited due to the small size of coronaries, especially in very young children (16), and their fast heart rates whereby the “quiet” period with relatively limited vessel motion limits the duration of the imaging window (17). This technique enables endogenous contrast enhancement without an exogenous contrast agent (18). Previous studies demonstrated that whole-heart 3D SSFP MRCA allowed visualization of the coronary arteries without the application of contrast agents in adults and children (2, 19, 20). Some hospitals use paramagnetic contrast agents to increase the contrast-to-noise ratio (CNR) and signal-to-noise ratio (SNR) of MRCA (5, 21, 22). Zagrosek et al. (21) compared the values of contrast-enhanced and noncontrast-enhanced 3D SSFP MRCA sequences in 21 adults. In their study, SNR of all the coronary arteries and CNR of the right coronary artery (RCA) increased after the application of gadolinium-diethylenetriaminepentaaceticacid (DTPA); the contrast-enhanced sequence did not increase the number of visible coronary segments nor image quality. It is uncertain whether the results in an adult group can apply to children. The aim of this study was to determine whether adding a gadolinium-based contrast agent to a 3D SSFP sequence increased the visibility of the origin, major branches, and proximal courses of coronary arteries in children.

## 2. Materials and methods

### 2.1. Study population

Ninety-two patients were referred for cardiac MR scans. Among the 92 patients, 2 were neonates, 3 had severe kidney problems, and 8 failed MR scans. They were excluded. Finally, the study enrolled seventy-nine patients (53 boys, 26 girls) from 1 month to 18 years old. They were referred for clinical evaluation of congenital heart disease (45 cases), cardiomyopathy (13 cases), myocarditis (1 case), Kawasaki disease (4 cases), cardiac tumor (5 cases), arrhythmia (8 cases), right ventricular aneurysm (1 case), chest distress (1 case), and syncope (1 case) by MRI. The Ethics Committee of Children's Hospital of Fudan University approved this study. All the patients’ parents or legal guardians gave written informed consent before the MR scan. Patients younger than 5 years needed sedation with oral chloral hydrate (0.5 ml/kg). To assess the image performance before and after contrast agent application for different ages, patients were classified into three groups: group 1 included patients aged 2 years or younger (*n* = 19), group 2 included patients aged 2–5 years (*n* = 17), and group 3 included patients older than 5 years (*n* = 43).

### 2.2. MRCA protocol

MRCA was performed with a 1.5-T MR unit (Magnetom Avanto, Siemens Medical Solutions, Erlangen, Germany). A 16-channel body coil was applied. Patients were placed supine on the magnet.

The cine MR scan in a four-chamber view was used to determine the optimal trigger delay time and acquisition window. These assessments were aided by evaluating the movement of the right coronary artery. The delay times derived from the first and last cardiac rest images were recorded as *T*_first_ and *T*_last_, respectively, and the optimal data acquisition window was calculated as *T*_opt_ = *T*_last_ − *T*_first_. In our research, it ranged from 28 to 234 ms in either end-systole or mid-diastole individually according to the heart rates of the patients. The trigger delay time was calculated as *T*_trig_ = *T*_first_ − 150 ms (23).

A 3D SSFP sequence (repetition time of 293.73–336.48 ms, echo time of 1.59–1.66 ms, 90° flip angle, 1 mm slice thickness, 164–380 × 250–420 mm field of view, 320 × 158–218 acquisition matrix, 1 number of averages) was used to obtain the coronary images. The images were collected at the end-expiratory of free breathing using respiratory navigation. The navigation bar was placed above the right diaphragm (2). The navigator acceptance window was ±2 mm. A second scan was applied using the same sequence after applying gadolinium-DTPA (Omniscan, GE Healthcare, Ireland) by bolus intravenous injection. The contrast agent dose was 0.2 mmol/kg.

### 2.3. Image analysis

Two blinded radiologists with more than 5 years of experience in MRCA independently assessed the image quality of the coronary arteries in random order. The images were reviewed by one of the two readers 1 month later. In addition to planar images, we used a commercial workstation (Advantage Workstation 4.5, GE Healthcare) to generate 3D images to better evaluate coronary anatomy. Disagreement was discussed before the final decision. The image quality was scored for the left main coronary trunk (LMT), the left anterior descending coronary artery (LAD), the left circumflex coronary artery (LCX), and the RCA according to a five-point scale (24): 0 = no coronary segments identified, 1 = coronary course uncertain, 2 = substantial blurring but initial coronary artery course traceable, 3 = coronary course readily traced with mild motion artifacts, and 4 = clearly visible. Moreover, the visible side branches were recorded for each case.

The lengths of visualized coronary arteries were measured on a workstation (Advantage Workstation 4.5, GE Healthcare) with the distance measurement tool (23).

SNR and CNR were calculated. Regions of interest (ROIs) of the blood signal intensity (SI_blood_) were placed in the lumen of the proximal coronary arteries. The myocardial signal intensity (SI_myo_) was measured from the myocardium close to the coronary vessel. The SD of background signal intensity (SD_noise_) was considered the mean signal intensity of three different ROIs (area≥20 cm^2^) outside the body. The formulas for calculating SNR and CNR are as follows: SNR = SI_blood_/SD_noise_, CNR = (SI_blood_ − SI_myo_)/SD_noise_ (23).

### 2.4. Statistical analysis

The quantitative values were presented as median values with 95% CIs. The detection rates of coronary arteries and side branches between pre- and postcontrast groups were compared by McNemar's *χ*^2^ test. The Wilcoxon signed-rank test was used to assess the differences in the results between each group. The intra- and interobserver agreements were evaluated with the weighted kappa test (for image quality) or intraclass correlation coefficient (ICC) test (for length, SNR, and CNR of the coronary artery). A *P* value <0.05 was considered significant.

## 3. Results

Of the 76 coronary arteries in group 1, 51 (67.1%) were visualized by noncontrast-enhanced MRCA and 71 (93.4%) were visualized by contrast-enhanced MRCA (image quality ≥ 1) (*P* = 0.000). In group 2, noncontrast-enhanced and contrast-enhanced MRCA allowed visualization of 66 of the 68 (97.1%) coronary arteries (*P* = 1.000). Of the 172 coronary arteries in group 3, noncontrast-enhanced and contrast-enhanced MRCA revealed 168 (97.7%) and 166 (96.5%) coronary arteries, respectively (*P* = 0.625).

Eighty side branches of coronary arteries were revealed in our study. They were more frequently observed on contrast-enhanced scans than noncontrast-enhanced scans in groups 1 (*P* = 0.045) and 2 (*P* = 0.008), but the detection rates between contrast-enhanced scans and noncontrast-enhanced scans did not reach statistical significance in group 3 (*P* = 0.317) (Table 1).

**Table 1:** Detected side branches of coronary arteries before and after the application of gadolinium-DTPA.

Side branches	Precontrast	Postcontrast	*P* value
Group 1 (*n* = 19)			
Diagonal	1	5	
Right conal	1	1	
Total	2	6	0.045
Group 2 (*n* = 17)			
Diagonal	4	7	
Obtuse marginal	2	2	
Right conal	7	9	
Acute marginal	4	6	
Right posterior descending artery	1	1	
Total	18	25	0.008
Group 3 (*n *= 43)			
Diagonal	9	9	
Left conal	1	1	
Anterior septal	1	1	
Obtuse marginal	7	7	
Right conal	11	12	
Right anterior ventricle	4	4	
Acute marginal	13	13	
Right posterior descending artery	1	2	
Total	47	49	0.317

**Figure 1:**
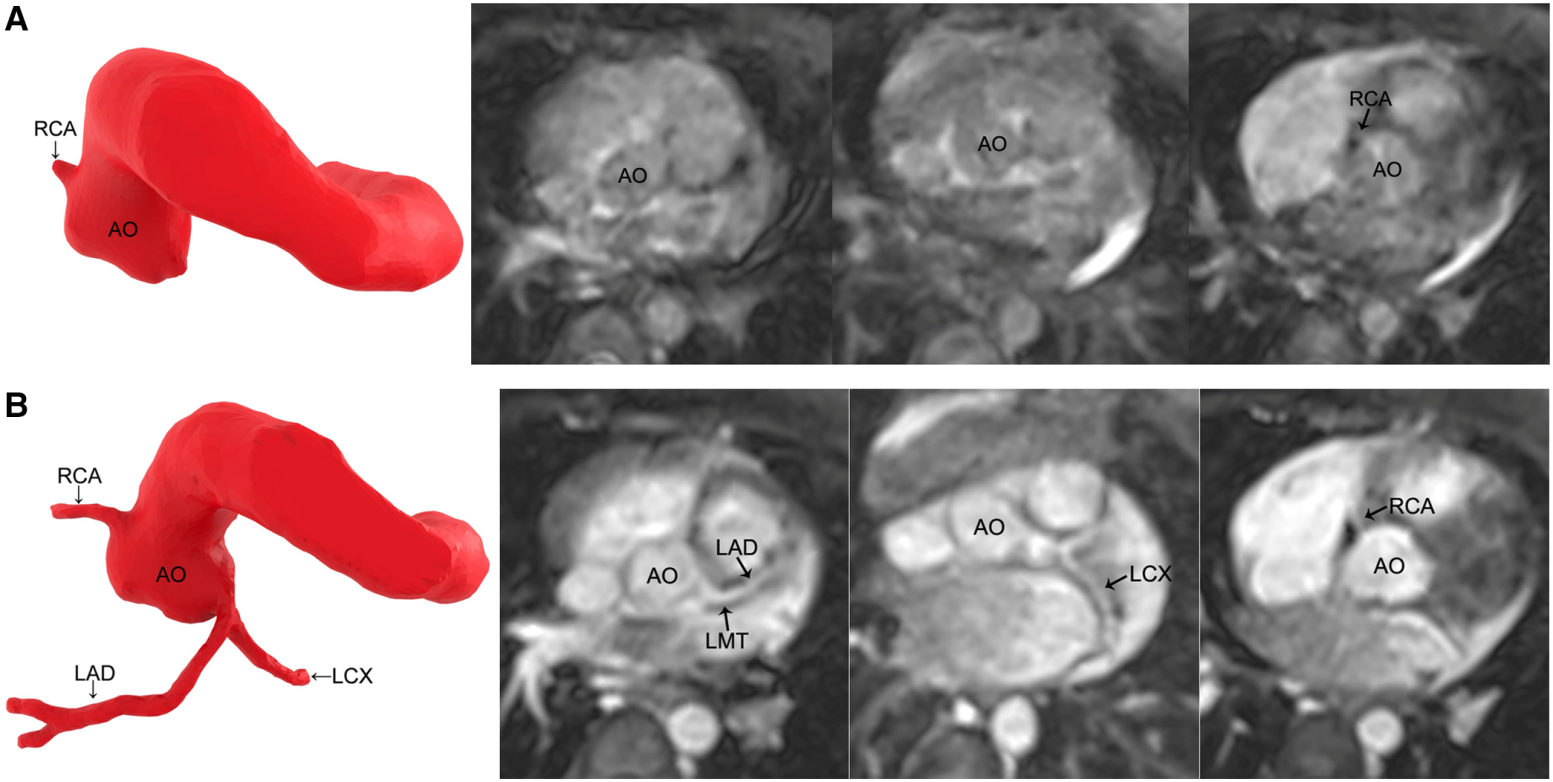
MR coronary angiography in a 7-month-old boy. (**A**) Noncontrast-enhanced MR coronary angiography only detects the origin of the RCA. (**B**) Contrast-enhanced MR coronary angiography images reveal all the coronary arteries. The image quality improves significantly. RCA, right coronary artery; AO, aorta; LMT, left main trunk; LAD, left anterior descending coronary artery; LCX, left circumflex coronary artery.

**Figure 2:**
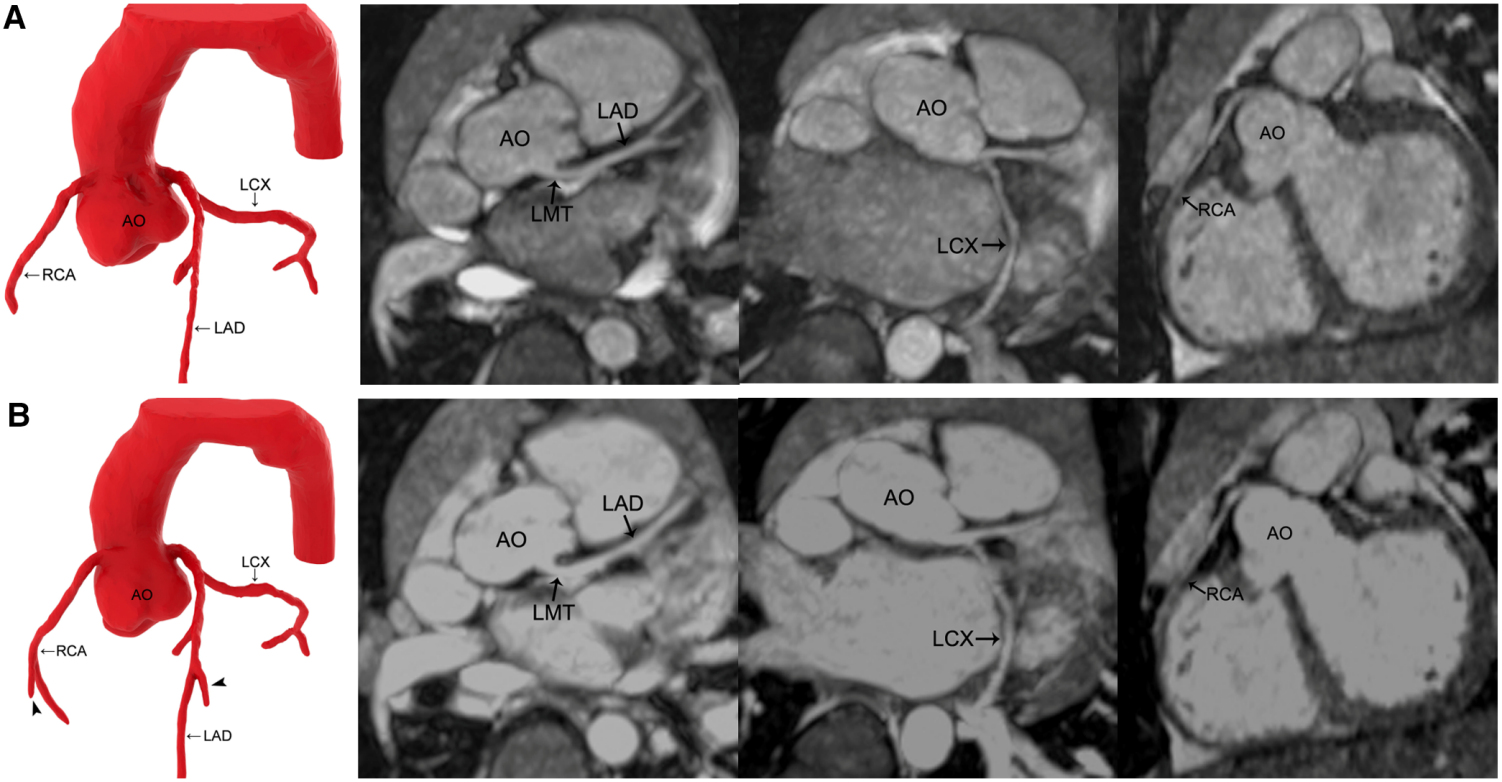
MR coronary angiography in a 3-year-old boy. (**A**) Precontrast imaging. (**B**) Postcontrast imaging. Compared with noncontrast-enhanced MR coronary angiography, the three dimension reconstruction image of MR coronary angiography after applying gadolinium-DTPA reveals more coronary artery side branches (arrowhead). Maximal intensity projection images show that the signal-to-noise ratio and contrast-to-noise ratio of all the coronary arteries increase after the application of gadolinium-DTPA, but the image quality is not improved significantly. AO, aorta; LMT, left main trunk; LAD, left anterior descending coronary artery; LCX, left circumflex coronary artery; RCA, right coronary artery.

**Figure 3:**
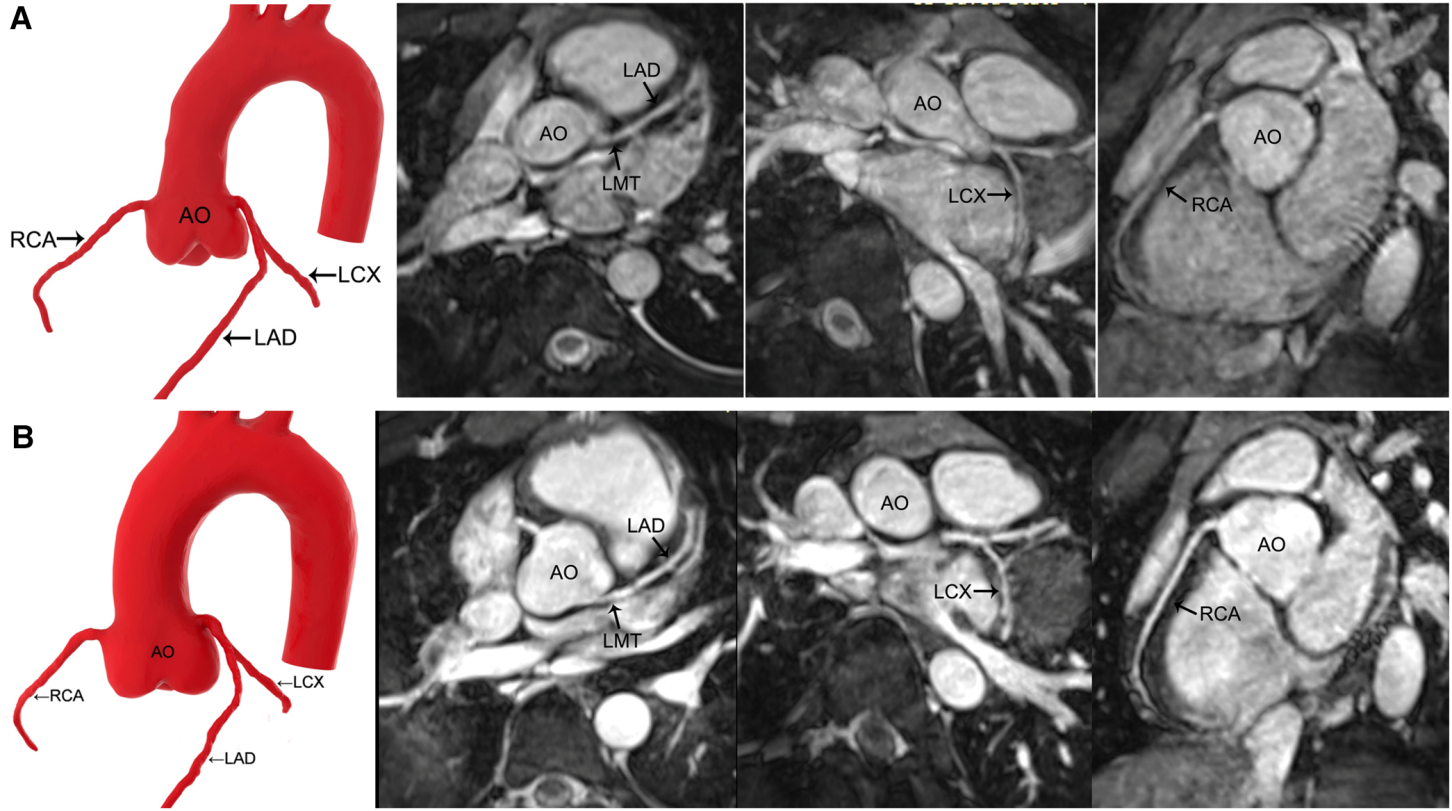
MR coronary angiography in a 13-year-old boy. (**A**) Precontrast imaging. (**B**) Postcontrast imaging. Despite an increase in the signal-to-noise ratio and contrast-to-noise ratio in the coronary arteries after the application of gadolinium-DTPA, contrast-enhanced MR coronary angiography neither improves the image quality significantly nor shows more side branches. AO, aorta; LMT, left main trunk; LAD, left anterior descending coronary artery; LCX, left circumflex coronary artery; RCA, right coronary artery.

The image quality in group 1 improved after gadolinium-DTPA injection (*P* < 0.05) (Figure 1). However, in groups 2 and 3, the image quality did not significantly improve after contrast agent application (*P* > 0.05) (Table 2) (Figures 2, 3).

**Table 2:** Image quality of the coronary arteries before and after the application of gadolinium-DTPA.

Vessel	Precontrast	Postcontrast	*P* value
Group 1 (*n* = 19)			
LMT	1 (0.87–2.39)	3 (1.93–3.34)	0.002
LAD	1 (0.75–2.30)	3 (2.06–3.31)	0.001
LCX	2 (0.95–2.52)	2 (1.81–3.03)	0.008
RCA	2 (1.21–2.47)	2 (1.67–2.96)	0.013
Group 2 (*n* = 17)			
LMT	4 (2.79–4.08)	4 (2.92–4.08)	0.317
LAD	4 (2.71–3.92)	4 (2.80–3.95)	0.317
LCX	4 (2.63–3.75)	4 (2.99–3.76)	0.180
RCA	4 (3.00–3.87)	4 (3.11–3.89)	0.180
Group 3 (*n* = 43)			
LMT	3 (2.78–3.41)	4 (2.87–3.56)	0.225
LAD	3 (2.92–3.51)	4 (2.87–3.52)	0.740
LCX	3 (2.76–3.34)	3 (2.73–3.46)	0.769
RCA	3 (2.97–3.50)	4 (2.98–3.60)	0.830

LMT, left main trunk; LAD, left anterior descending coronary artery; LCX, left circumflex coronary artery; RCA, right coronary artery.

The length of LAD and LCX was longer in group 1, and the LCX length was longer in group 2 by contrast-enhanced MRCA (*P* < 0.05); there was no statistical significance of the differences between pre- and postcontrast groups in the other patients (*P* > 0.05) (Table 3).

**Table 3:** Length (mm) of the coronary arteries before and after the application of gadolinium-DTPA.

Vessel	Precontrast	Postcontrast	*P* value
Group 1 (*n* = 19)			
LMT	6.50 (4.76–7.06)	6.10 (4.26–6.88)	0.083
LAD	13.80 (9.51–27.57)	23.80 (17.68–32.07)	0.005
LCX	10.95 (7.17–20.88)	23.25 (15.52–34.02)	0.004
RCA	38.25 (22.70–48.63)	46 (31.19–53.89)	0.056
Group 2 (*n* = 17)			
LMT	9.00 (7.40–15.06)	10.70 (8.18–15.12)	0.248
LAD	24.90 (20.51–36.21)	27.50 (22.73–39.55)	0.055
LCX	46.90 (26.68–48.96)	48.70 (43.47–61.95)	0.023
RCA	66.30 (53.96–71.57)	66.90 (54.94–81.31)	0.234
Group 3 (*n* = 43)			
LMT	11.05 (10.52–15.31)	11.30 (10.82–16.06)	0.071
LAD	38 (36.28–55.26)	43.20 (39.39–53.55)	0.325
LCX	36.45 (34.95–50.19)	42.70 (38.57–54.53)	0.081
RCA	72.5 (67.63–88.62)	86.45 (70.06–91.72)	0.062

LMT, left main trunk; LAD, left anterior descending coronary artery; LCX, left circumflex coronary artery; RCA, right coronary artery.

The SNR and CNR of all the vessels in group 1 and group 2 and the LCX and RCA in group 3 improved after contrast agent injection (*P* < 0.05), whereas LMT and LAD remained unchanged in group 3 (*P* > 0.05) (Tables 4, 5) (Figures 2, 3).

**Table 4:** SNR of the coronary arteries before and after the application of gadolinium-DTPA.

Vessel	Precontrast	Postcontrast	*P* value
Group 1 (*n* = 19)			
LMT	23.60 (16.24–31.71)	33.45 (28.90–41.80)	0.008
LAD	22.45 (14.54–28.68)	30.10 (24.45–38.76)	0.026
LCX	20.48 (14.75–29.55)	32.24 (25.61–36.21)	0.012
RCA	15.78 (12.03–27.60)	28.32 (24.18–34.80)	0.007
Group 2 (*n* = 17)			
LMT	37.86 (31.97–50.22)	56.66 (50.70–69.27)	0.000
LAD	32.77 (27.91–41.08)	45.00 (40.32–55.30)	0.001
LCX	35.89 (29.58–40.71)	53.31 (43.52–61.29)	0.001
RCA	32.97 (25.85–41.46)	44.16 (36.26–53.35)	0.001
Group 3 (*n* = 43)			
LMT	48.81 (46.48–62.86)	57.96 (52.56–67.90)	0.134
LAD	43.66 (41.38–56.92)	47.85 (44.74–57.64)	0.751
LCX	39.39 (40.69–55.76)	47.43 (46.31–62.49)	0.012
RCA	41.57 (38.26–51.54)	50.83 (44.03–59.22)	0.042

SNR, signal-to-noise ratio; LMT, left main trunk; LAD, left anterior descending coronary artery; LCX, left circumflex coronary artery; RCA, right coronary artery.

**Table 5:** CNR of the coronary arteries before and after the application of gadolinium-DTPA.

Vessel	Precontrast	Postcontrast	*P* value
Group 1 (*n* = 19)			
LMT	10.33 (4.55–14.50)	16.26 (13.08–22.02)	0.006
LAD	8.29 (3.86–11.40)	13.36 (8.67–18.94)	0.041
LCX	8.63 (5.22–13.15)	13.93 (8.70–17.51)	0.023
RCA	3.13 (1.63–10.08)	16.98 (8.92–14.46)	0.004
Group 2 (*n* = 17)			
LMT	21.81 (13.92–27.02)	31.60 (26.13–38.87)	0.001
LAD	13.92 (9.50–18.24)	20.38 (14.96–25.69)	0.009
LCX	16.24 (10.04–19.39)	21.01 (18.77–31.07)	0.002
RCA	13.91 (7.46–18.68)	18.19 (10.66–24.41)	0.028
Group 3 (*n* = 43)			
LMT	24.58 (23.32–36.95)	30.80 (29.77–40.92)	0.084
LAD	19.71 (18.73–30.90)	23.53 (22.12–31.50)	0.234
LCX	18.01 (17.87–29.88)	20.06 (24.08–35.96)	0.002
RCA	19.17 (15.61–26.04)	28.46 (21.75–32.85)	0.004

CNR, contrast-to-noise ratio; LMT, left main trunk; LAD, left anterior descending coronary artery; LCX, left circumflex coronary artery; RCA, right coronary artery.

The intra- and interobserver agreements in this study were high (0.803–0.998) for image quality, length, SNR, and CNR of the coronary arteries in both pre- and postcontrast groups (Table 6).

**Table 6:** Intra- and interobserver agreements before and after the application of gadolinium-DTPA.

Vessel	Intraobserver agreement		Interobserver agreement	
	Precontrast	Postcontrast	Precontrast	Postcontrast
Image quality				
LMT	0.885	0.815	0.859	0.835
LAD	0.825	0.818	0.805	0.806
LCX	0.815	0.821	0.803	0.812
RCA	0.834	0.814	0.822	0.849
Length				
LMT	0.995	0.997	0.994	0.996
LAD	0.988	0.993	0.986	0.991
LCX	0.991	0.995	0.971	0.983
RCA	0.997	0.998	0.996	0.995
Signal-to-noise ratio				
LMT	0.911	0.917	0.898	0.873
LAD	0.922	0.919	0.903	0.890
LCX	0.898	0.904	0.834	0.880
RCA	0.921	0.923	0.886	0.896
Contrast-to-noise ratio				
LMT	0.907	0.880	0.881	0.895
LAD	0.918	0.885	0.883	0.884
LCX	0.901	0.908	0.855	0.887
RCA	0.911	0.902	0.889	0.878

LMT, left main trunk; LAD, left anterior descending coronary artery; LCX, left circumflex coronary artery; RCA, right coronary artery.

## 4. Discussion

Our study compared the visibility of coronary artery anatomy in the same child before and after applying gadolinium contrast using a 1.5-T 3D SSFP sequence. Although there is no gold standard, such as angiography or direct observation at the surgery for coronary anatomy, this limitation is mitigated to some degree as each child acts as its control.

In our study, the gadolinium contrast-enhanced 3D SSFP sequence improved not only SNR and CNR but also the image quality of coronary arteries in children under 2 years old and detected more coronary arteries than the noncontrast-enhanced 3D SSFP sequence. Moreover, the contrast-enhanced 3D SSFP sequence detected more coronary artery side branches in patients younger than 5 years. Because young children's coronary arteries are thin, adequate SNR and CNR imaging is critical for delineating coronary arteries. The application of an extracellular contrast agent is useful. The extracellular contrast agent in MRCA can enhance the blood signal due to its T1 shortening effect (18, 25). It is difficult to visualize the distal part of LAD and LCX due to their tortuous courses and small diameters in young children. In our study, the contrast-enhanced SSFP sequence displayed longer lengths for LAD in children under 2 years old and for LCX in children younger than 5 years. The increased CNR after injection of contrast agent proved very useful in delineating distal coronary artery segments in young children. Gadolinium contrast-enhanced MRCA increased SNR and CNR of most coronary arteries in children over 2 years old, but it did not improve the image quality significantly in our study, as reported in a previous study in adults (21).

Our results would help to improve proper MRCA protocols for children of different ages. Gadolinium-DTPA is widely used in our hospital; this contrast agent is contraindicated in patients with severe kidney problems (26, 27). For such patients older than 5 years, the noncontrast-enhanced 3D SSFP sequence is qualified to assess the morphology of coronary arteries without the nephrotoxic and neurotoxic risks. For patients older than 2 years to 5 years, the application of gadolinium-DTPA detects more coronary artery side branches, although it does not improve the image quality significantly. We recommend the contrast-enhanced 3D SSFP sequence for patients without the contraindications of gadolinium-DTPA application in this age range; the noncontrast-enhanced 3D SSFP sequence is an alternative to depict the main trunk of coronary arteries for patients with the contraindications of gadolinium-DTPA application. For patients younger than 2 years without the contraindications of gadolinium-DTPA application, the contrast-enhanced 3D SSFP sequence is necessary to improve image performance. For patients younger than 2 years with the contraindications of gadolinium-DTPA application, MRCA is not recommended.

In addition to the contrast agent, we took several measures to improve the image performance. For the 3D SSFP sequence, we used a long repetition time (293.73–336.48 ms) to increase the SNR. Effective sedation for children younger than 5 years and respiratory training for children older than 5 years before the MR scan provided regular breathing. Thus, acceptable navigator efficiency was increased and data acquisition time and respiratory motion artifacts were reduced. The trigger delay time and acquisition window were decided individually for each child according to the longest cardiac rest period (3). For children with low heart rates, the images were acquired during mid-diastole. With the increase in heart rates, the end-systolic rest period tended to be longer than the diastolic rest period (6, 17). Therefore, end-systole was preferred by the technician in children with high heart rates.

Despite the application of gadolinium-DTPA and the above measures, the image performance in children younger than 2 years was still unsatisfactory. It was even worse than precontrast imaging in children older than 2 years in our study. The number of cases in our study is too small, and this result may not be universal. However, it is really a difficult point to clearly show the coronary arteries of young populations. One improved solution is the use of intravascular contrast agents. Intravascular contrast agents have much greater T1 relaxivity than extracellular contrast agents, and they keep a high concentration in the blood and also have fewer exosomes into the myocardium (18, 28). These characteristics of intravascular contrast agents enable them to obtain high-quality vascular-enhanced images. In addition to intravascular contrast agents, extracellular contrast agents with high T1 relaxivity are also considered because they may be more available than intravascular contrast agents. For example, gadobutrol is a nonspecific extracellular magnetic resonance contrast agent with the highest T1 relaxivity in plasma among gadolinium-based contrast agents (29–31). Therefore, these contrast agents may perform better in coronary artery imaging in young children. Another alternative imaging modality is CT coronary angiography. Despite ionizing radiation, CT coronary angiography provides excellent spatial resolution, which is required to assess small coronary arteries in children. Furthermore, simple preparation and short investigation time make CT coronary angiography more suitable for young children and critically ill patients. CT coronary angiography can provide direct anatomical detail of the coronary arteries. With the advancements in technology, dose estimates of CT coronary angiography are in a rather low range. The benefit-to-risk ratio of CT coronary angiography has been greatly improved recently, so the technique may be considered a potentially noninvasive diagnostic method (32). 1.5-T 3D SSFP MRCA and CT coronary angiography are complementary imaging modalities and may have different indications.

## 5. Limitations

One limitation is the relatively small sample size of groups 1 and 2, which may have limited the statistical power of the evaluation. Despite the weaknesses, our results preliminarily indicate the different MRCA image performance with and without contrast agent application in children of different age groups.

## 6. Conclusions

The use of gadolinium contrast in combination with the 3D SSFP sequence is necessary for coronary imaging in children under 2 years of age, and it might be helpful in those between 2 and 5 years; however, we have not seen that the use of gadolinium contrast can significantly improve the quality of the information about the coronary arteries in children older than 5 years.

## Data Availability

The original contributions presented in the study are included in the article, further inquiries can be directed to the corresponding authors.
